# *Helicobacter pylori*, Republic of Georgia

**DOI:** 10.3201/eid1105.040755

**Published:** 2005-05

**Authors:** Katrina Kretsinger, Jeremy Sobel, Nato Tarkhashvili, Neli Chakvetadze, Marina Moistrafishvili, Merab Sikharulidze, Ben D. Gold, Marina Chubinidze, Paata Imnadze

**Affiliations:** * Centers for Disease Control and Prevention, Atlanta, Georgia, USA;; † National Center for Disease Control, Tbilisi, Republic of Georgia;; ‡ Emory University School of Medicine, Atlanta, Georgia, USA

**Keywords:** prevalence, *Helicobacter pylori*, Georgia (Republic of), social class, stomach neoplasms

**To the Editor:** *Helicobacter pylori* infection is a principal cause of chronic active gastritis and peptic ulcer disease as well as gastric adenocarcinoma and mucosa-associated lymphoid tissue lymphoma (1). Poverty and crowding have been associated with infection epidemiologically (2,3). The Republic of Georgia has a per capita annual income of US $591, making it one of the poorest countries in the world (4,5). Georgia also reports a high annual incidence of gastric cancer, 17/100,000 population in 2002 (National Center for Disease Control, Tbilisi, unpub. data), which suggests an elevated prevalence of *H. pylori* infection. Testing and treatment for *H. pylori* are not practiced in this country, and diagnostic capacity for *H. pylori* is nonexistent. In October 2003, we conducted an exploratory pilot study of *H. pylori* infection to begin characterizing prevalence and risk factors for infection.

We studied a convenience sample of adults residing in or near the capital city of Tbilisi. Urban participants were recruited in 11 of Tbilisi's 12 residential districts and in 1 district of Rustavi, a city 20 miles south of Tbilisi. Rural participants were recruited from 3 villages within 10 miles of Tbilisi. In each district or village, we nonsystematically selected 10 households and recruited 1 adult per household. Exclusion criteria included age <18 years; reported allergy to omeprazole, clarithromycin, or amoxicillin; or treatment with any antimicrobial agent within the preceding 2 weeks. This protocol was reviewed and approved by the Human Subjects Review Board at the National Center for Disease Control, Tbilisi. Active infection with *H. pylori* was measured by a validated, point-of-care ^13^C-urea breath test (Meretek Corporation, Lafayette, CO, USA) (6). Participants responded to a questionnaire that requested information about gastrointestinal symptoms during the preceding 12 months; diagnosis of gastritis, peptic ulcer disease, and gastric cancer made by a physician; family history of peptic ulcer disease or gastric cancer; and knowledge about *H. pylori*. Low, medium, or high socioeconomic status categories were designated on an ecologic basis, according to average real estate prices and common perception of the living standard of the participant's district or village of residence. Analyses were conducted with SAS (SAS Institute, Cary, NC, USA) version 9.0. Measures of inference are not reported because participants did not constitute a rigorously selected population sample.

Of 136 persons eligible to participate in the study, 135 (99%) consented to take part. Median age was 39 (range 19 – 79); 82 participants (61%) were women. Twenty-seven (20%) reported having some knowledge of *H. pylori*, but none had been tested or treated for the infection. Ninety-seven (72%) participants had active *H. pylori* infection: 58 (71%) of 82 women and 39 (74%) of 53 men. Thirty (77%) of 39 participants ≥50 years of age tested positive for *H. pylori* compared to 67 (70%) of 96 participants <50 years of age.

Seventy-one (84%) of 85 participants residing in neighborhoods of low-socioeconomic status were infected versus 26 (52%) of 50 participants residing in neighborhoods of medium- or high-socioeconomic status (crude prevalence odds ratio 4.68). Twenty-three (85%) rural participants were infected compared to 74 (69%) of 108 urban participants (crude prevalence odds ratio 2.64).

Gastrointestinal symptoms were common, but did not correlate with active infection. One hundred five participants (78%) reported recurrent epigastric pain within the past year; and 120 participants (89%) reported a variety of gastrointestinal symptoms, including epigastric pain. Persons with active infection were no more likely to report epigastric pain (75 [78%] of 97) than persons without infections (30 [79%] of 38). One participant reported gastric cancer and was *H. pylori*–positive. Seven participants reported a family history of gastric cancer; all were *H. pylori*–positive. Five participants, of whom 3 were *H. pylori*–positive, reported a history of gastric surgery for peptic ulcer disease. More rural participants (23 of 27) reported a history of ruptured ulcers or a family member with gastric cancer than did urban participants (74 of 108). The frequency of reported gastrointestinal symptoms was similar between urban and rural participants.

To our knowledge, this is the first survey of *H. pylori* infection in Georgia. We found a high rate of infection with *H. pylori*. Participants also reported a very high rate of dyspeptic symptoms, although these were not correlated with infection. This small convenience sample survey has several limitations, however. First, our participants did not constitute a systematically selected population sample. Second, rural populations were underrepresented. Finally, we used neighborhood or village of residence as a marker for socioeconomic status without specific income information from the participants. Therefore, socioeconomic status misclassification possibly occurred and the association between infection and socioeconomic status may not be accurate.

Nevertheless, it is unlikely that we substantially over- or underrepresented infection prevalence in the general population. Despite the limitations of this study, our results clearly indicate that *H. pylori* is a serious public health problem in Georgia. There is a pressing need to educate medical professionals and the general public about *H. pylori* infection and gastrointestinal illness and to introduce diagnosis of this infection and appropriate treatment for it into standard medical practice. In addition, rigorous population surveys that include children are needed to identify high-risk groups of persons for targeted public health interventions (7).
